# Bibliographic Analysis of *Nature* Based on Twitter and Facebook Altmetrics Data

**DOI:** 10.1371/journal.pone.0165997

**Published:** 2016-12-01

**Authors:** Feng Xia, Xiaoyan Su, Wei Wang, Chenxin Zhang, Zhaolong Ning, Ivan Lee

**Affiliations:** 1 School of Software, Dalian University of Technology, Dalian 116621, P. R. China; 2 School of Information Technology and Mathematical Sciences, University of South Australia, Adelaide, SA 5095, Australia; Beihang University, CHINA

## Abstract

This paper presents a bibliographic analysis of *Nature* articles based on altmetrics. We assess the concern degree of social users on the *Nature* articles through the coverage analysis of Twitter and Facebook by publication year and discipline. The social media impact of a *Nature* article is examined by evaluating the mention rates on Twitter and on Facebook. Moreover, the correlation between tweets and citations is analyzed by publication year, discipline and Twitter user type to explore factors affecting the correlation. The results show that Twitter users have a higher concern degree on *Nature* articles than Facebook users, and *Nature* articles have higher and faster-growing impact on Twitter than on Facebook. The results also show that tweets and citations are somewhat related, and they mostly measure different types of impact. In addition, the correlation between tweets and citations highly depends on publication year, discipline and Twitter user type.

## Introduction

Activities on social media have been an emerging approach to evaluate the early impact of scholarly publications, and studies based on Twitter [1–3], Researchgate [4, 5], web CV [6, 7] and so on have been conducted in literature. As a generalization of article level metrics, *altmetrics* can assess the popularity or social impact of publications based on data collected by social media platforms [8, 9]. Compared with traditional citation-based metrics, altmetrics can reduce the delay for accumulation and cover new forms of scholarly content (e.g., datasets, software, and research blogs) to achieve broader, more diversiform and rapid impact analysis [10–12]. Therefore, altmetrics are becoming increasingly important as researchers, academic institutions and funders look for new ways to track the impact of research outputs in real time.

Although the study of altmetrics is still in the early stage, significant research has already been done. So far, most of the studies have focused on the representativeness and validity of social media platforms as a source of impact assessment. For instance, Thelwall et al. [13] compared 11 altmetrics with Web of Science (WoS) citations for PubMed articles with at least one altmetrics mentioned in each case. They found that the coverage of all the altmetrics except for Twitter seems to be low, and thus it is not clear whether they are prevalent enough to be used in practice. Zahedi et al. [14] analyzed the presence and possibilities of altmetrics for bibliometric and performance analysis based on 20,000 random publications from the WoS. Wouters and Costas [15] presented a comprehensive assessment of limitations and strengths of the most current novel impact monitors including webometrics and altmetrics. They concluded that these new tools seem to be more useful for self-analysis than for systematic impact measurement at different levels of aggregation. Based on a comprehensive dataset from very disparate sources, Bornmann [16] studied the validity of altmetrics data for measuring societal impact. One promising result of this study is that Altmetric data seem able to indicate the papers which produce societal impact, but it is not clear which kind of impact is measured. Haustein et al. [17] investigated the use and coverage of social media environments amongst a sample of bibliometricians examining both their own use of online platforms and the use of their papers on social reference managers. They found 82% of articles published by the sample bibliometricians were included in Mendeley libraries.

Existing studies such as the ones mentioned above face major limitations that all of them ignore the influence of journal, discipline and time on the validity of altmetrics. Considering the influence of discipline, Hammarfelt [18] analyzed the altmetric coverage and impact of the humanities-oriented articles and books published by Swedish universities during 2012. He found that Mendeley has the highest coverage of journal articles followed by Twitter while very few of the publications are mentioned in blogs or on Facebook. In addition, he argued that altmetrics could evolve into a valuable tool for assessing research in the humanities. Instead of focusing on one discipline, we conduct a multi-disciplinary study by analyzing the distribution of *Nature* articles on social media by publication year and discipline. Moreover, our research investigates altmetrics from the two most popular social media platforms, Twitter and Facebook.

Some other studies have focused on the correlation between citations and various social media event counts to determine whether both types of metrics measure similar concepts. For instance, Xin Shuai et al. [19] analyzed the online response to the preprint publication of a cohort of 4,606 scientific articles submitted to the preprint database arXiv.org, and they found Twitter mentions is better to predict citations than arXiv downloads. However, they do not consider the influence of scientific fields on the correlation. For the biomedical literature, Haustein et al. [20] analyzed their tweets and citations based on a set of 1.4 million documents covered by both PubMed and WoS and published between 2010 and 2012. They found there is low correlation between tweets and citations, and argued that Twitter-based indicators reflect another kind of impact not comparable to traditional citation indicators for the biomedical literature. Nevertheless, they ignore the influence of different journals. Through mining all the tweets between July 2008 and November 2011 containing links to articles in the Journal of Medical Internet Research, Eysenbach et al. [21] found there are strong correlations between tweets and citations, and the collective intelligence of Twitter users can predict citations with limitation. This confirms that the correlation should be analyzed based on a specific journal. However, they just focus on a specific discipline, and do not analyze the correlation of different disciplines in a comprehensive scientific magazine. Through the analysis of article-level metrics of 27,856 PLOS ONE articles, De Winter [22] concluded that the scientific citation process acts relatively independently of the social dynamics on Twitter. Based on a set of 1,589,440 publication records downloaded from Altmetric.com, Costas et al. [23] presented an extensive analysis of the presence of different altmetric indicators provided by Altmetric.com across scientific fields.

Nevertheless, these existing studies do not account for the publication year and the role of social users. Our work differs from these existing researches in that it analyzes the correlation between tweets and citations for *Nature* articles by publication year, discipline and Twitter user type. In particular, we think different social media users have different concerns for research topics. And the research for user type can help to explore the detailed correlation between citations and social media. To the best of our knowledge, the correlation between citations and tweets has not been studied for *Nature* articles by publication year, discipline and Twitter user type in existing researches about altmetrics.

Altmetrics introduce a new perspective on the research activity, relating research impact and social skill. This makes possible the early assessment of academic influence and development of public-access rankings. Following this idea, this work explores the validity of altmetrics (Twitter and Facebook) and relationship between altmetrics and traditional metric (citation) to make clear the meaning of these metrics and their interactions with citation. Focusing on Twitter and Facebook, we present a bibliographic analysis of *Nature* articles. As a famous comprehensive British scientific magazine, *Nature* was founded in 1869 and it is one of the oldest and authoritative scientific journals in the world. We firstly study the distribution of *Nature* papers on Twitter and Facebook through the coverage and mention rate analysis by publication year and discipline. This enables us to determine which social media platform develops more rapidly over time and which discipline draws more attentions from social media. Moreover, we discuss the relationship between citations and tweets for *Nature* articles by publication year, discipline, and Twitter user type to explore whether both types of metrics measure similar concepts. We also evaluate the influential discipline and research topic in *Nature* from the perspective of both altmetrics and citations. This will help to explore altmetrics indicators as complements to traditional metrics in research evaluation.

## Methods

### Data

We have downloaded the metadata for all *Nature* research papers from the online literature database over the period between January 2010 and June 2015, including title, publication date, discipline, doi, and keywords. It should be noted that the data about the publications from January 2015 to June 2015 are used to analyze the year-round impact tendency of *Nature* articles published in 2015, although the data in the full year cannot be obtained.

In order to assess the impact based on altmetrics and citations, we have crawled the accumulated number of tweets and Facebook posts from nature.altmetric.com and citations from the Web of Science which is one of the most comprehensive citation repositories in the world, in June 2015. We combine these two data sets by doi of paper. As shown in Table 1, it is the statistics of *Nature* publications in the data set, and 4276 articles are used in total for our analysis. Note that, for a specific *Nature* article, it may belong to multiple disciplines, and thus the sum of article number for four disciplines is greater than 4276. In order to carry out the bibliographic analysis by Twitter user type, we utilize the user type classification given in Altmetric.com (http://support.altmetric.com/knowledgebase/articles/435434-how-are-twitter-demographics-determined). Altmetric categorizes users based on information (keywords in profile descriptions, the types of journals that users link to, and follower lists) in users’ profiles on Twitter. Twitter users are divided into the following four types based on the information in their profiles on Twitter:

- **Member of the public**: someone who does not link to scholarly literature and does not fit any of the categories below.
- **Scientist**: someone who is familiar with the literature.
- **Practitioner**: a clinician or researcher who is working in clinical science.
- **Science communicator**: someone who links frequently to scientific articles from a variety of different journals or publishers.

**Table 1:** Statistics of *Nature* publications in the data set.

	2010	2011	2012	2013	2014	2015	Total
Biology Sciences	366	576	572	557	560	254	2876
Chemical Sciences	101	161	132	25	32	22	473
Earth & Environment Sciences	78	113	112	96	95	32	526
Physical Sciences	113	146	171	160	165	76	831
Total	552	789	846	846	842	401	4276

### Analysis Methods

In order to evaluate the representativeness and validity of Twitter and Facebook as data sources for altmetrics, we analyze the distribution of academic information about *Nature* articles on Twitter and Facebook. Some researches [24, 25] used coverage and mention rate to do distribution analysis. Here, we give the definitions of the metrics used in our distribution analysis.

**Definition 0.1** ***Twitter(T)/Facebook(F) Coverage*** Cov^n^ *is defined as the proportion of articles tweeted/posted at least once*, *i.e.*,

$${\text{Cov}}^{n}=\frac{N^{n}}{N}\;n\in \left\{T,F\right\}$$ (1)

*where* *N* *is the total amount of articles for the analysis*, *and* *N*^n^ *is the amount of articles tweeted/posted at least once*.

**Definition 0.2** ***Twitter(T)/Facebook(F) Mention Rate*** MR^n^ *is defined as the mean number of tweets per tweeted/posted paper*, *i.e.*,

$${\text{MR}}^{n}=\frac{\sum_{i=1}^{N}C_{i}^{n}}{N^{n}}\;n\in \left\{T,F\right\}$$ (2)

*where* $C_{i}^{n}$ *is the tweeted/posted count of the paper i*.

The coverage is used to evaluate the concern degree of social users on a *Nature* article and the development of the social media platform on the academic field, while the mention rate is used to examine the impact of a *Nature* article on a social media platform. In this paper, we assess the concern degree of Twitter users and Facebook users on *Nature* articles and the impact of articles on Twitter and Facebook by publication year and discipline. For Twitter, we consider the influence of user types on the concern degree of social users and the social impact. Moreover, the top fifteen frequently tweeted articles and the top fifteen frequently mentioned articles on Facebook are listed to explore the scholarly focus of Twitter and Facebook.

We also analyze the relationship between tweets and citations for *Nature* publications to determine whether both types of metrics measure similar concepts. The citations of articles published in 2015 have a big potential changes in the next few years because citation needs time to accrue. Therefore, the correlation between tweets and citations for articles published in 2015 can be disturbed by the low citation, thus we only use the data over the period between 2010 and 2014 in our relationship analysis. Since the relationship may be influenced by a variety of factors including publication date, discipline and Twitter user type, we evaluate the Spearman correlation between tweets and citations for the *Nature* articles by these factors.

## Results and Discussion

### Bibliographic Analysis Based on Twitter and Facebook

#### Twitter and Facebook Coverages

In order to carry out the bibliographic analysis of *Nature* articles based on Twitter and Facebook, we assess the coverage and mention rate of Twitter and Facebook for *Nature* articles. Here, we first investigate the coverages of Twitter and Facebook for *Nature* articles by publication year to evaluate the concern degree of social users on *Nature* articles published in different years.

**Fig 1:**
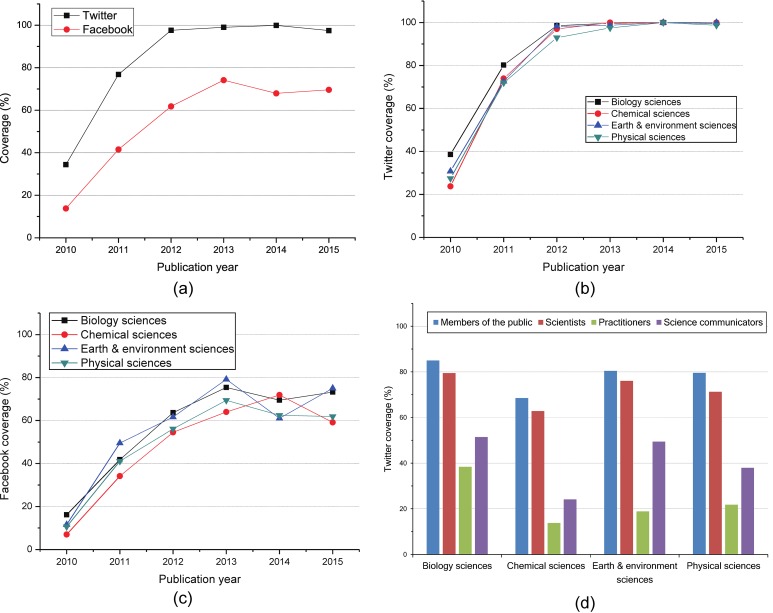
Facebook and Twitter coverage. (a): Coverage comparison between Twitter and Facebook. (b): Twitter coverage by publication year and discipline. (c): Facebook coverage by publication year and discipline. (d): Twitter coverage by user type and discipline.

In Fig 1, we show the coverages of Twitter and Facebook for *Nature* articles by publication year, discipline and Twitter user type. Fig 1(a) shows the Tcoverages of witter and Facebook for *Nature* articles published in different years. We can find that both Twitter users and Facebook users are interested in a few *Nature* articles published in 2010, where the Twitter coverage is no more than 35% and the Facebook coverage is less than 14%. As Twitter and Facebook evolve, social users increasingly focus on the scholarly documents, thus the coverages of Twitter and Facebook show an increasing trend over the publication time. For *Nature* articles published in 2013, the Twitter coverage approaches 100%, and the Facebook coverage is up to 75%. Moreover, the coverages of Twitter and Facebook are relatively stable for *Nature* articles published after 2013. Note that, Twitter consistently exceeds Facebook in terms of coverage. Maybe the Twitter users focus more on academic information. We can conclude that Twitter develops more rapidly than Facebook in academic field.

In order to assess the concern degree of social users on *Nature* articles from different disciplines and determine which discipline develops more rapidly on social media platform, we analyze the Twitter coverage and the Facebook coverage by publication year and discipline. Fig 1(b) and 1(c) illustrate the coverages of Twitter and Facebook by publication year and discipline. We can see that, for all disciplines, both Twitter coverage and Facebook coverage show an increasing trend over the published time. For *Nature* articles published in 2010 and 2011, Twitter coverage of biology sciences is significantly higher than those of other disciplines and Twitter coverages of other three disciplines show a similar lower growth trends. For *Nature* articles published after 2012, Twitter coverage of all disciplines approaches 100%. Compared with the Twitter coverage, the differences of Facebook coverage among distinct disciplines are relatively larger. For the articles which are not published in 2014, the Facebook has a lower coverage for chemical sciences than other disciplines and a relatively higher coverage for biology sciences and earth & environment sciences. For *Nature* articles published in 2014, we can also see there is a great change to the Facebook coverage with chemical sciences enjoying highest value and earth & environment sciences having the lowest value.

According to the category of Twitter user types given in Altmetric.com, we investigate Twitter coverage by user type and discipline based on the *Nature* articles published from 2010 to 2015 to assess the concern degree of different Twitter user types on *Nature* articles for different disciplines. Fig 1(d) shows the Twitter coverage by user type and discipline. We can find that for all disciplines, members of the public have the highest concern degree, and then scientists, science communicators, and practitioners. We can also see that members of the public and scientists have the similar concern degree on all disciplines and the coverages are greater than 60 percent. For science communicators, they are more interested in biology sciences and earth & environment sciences than the rest. Practitioners have the greatest concern degree on biology sciences among all disciplines.

#### Twitter and Facebook Mention Rates

**Fig 2:**
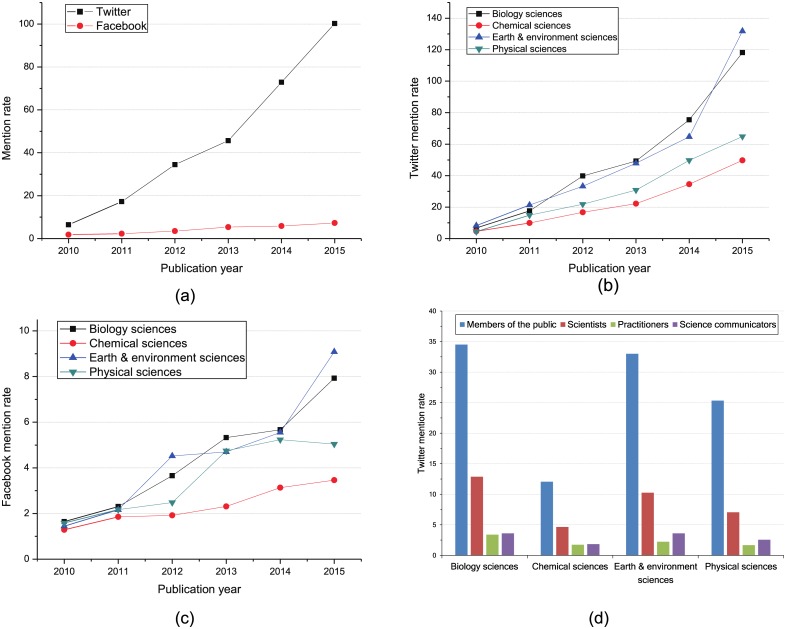
Facebook and Twitter mention rate. (a): Mention rate comparison between Twitter and Facebook. (b): Mention rate comparison between Twitter and Facebook. (c): Facebook mention rate by publication year and discipline. (d): Twitter mention rate by user type and discipline.

Besides concern degree, we also study the social impact of *Nature* articles on Twitter and Facebook by evaluating the Twitter and Facebook mention rates for *Nature* articles published in different years. Fig 2 shows Twitter and Facebook mention rates for *Nature* articles by publication year, discipline and Twitter user type. Fig 2(a) gives the mention rates of Twitter and Facebook for *Nature* articles published in different years. We can see that there is a continuous growth for both Twitter and Facebook mention rates. For *Nature* articles published from 2010 to 2015, Twitter mention rate increases from 6.5 to 100.2. In comparison, Facebook mention rate rises by merely 5.5 over the same period. Thus, we can conclude that the *Nature* articles attract more attention from Twitter than Facebook.

Unlike some previous studies [21, 26], we consider the discipline of papers. We analyze the mention rates of Twitter and Facebook by publication year and discipline to evaluate the social impact of the *Nature* articles on different disciplines that are published in different years. Fig 2(b) and 2(c) show the mention rates of Twitter and Facebook by publication year and discipline. It can be found that there is an ascending trend of both Twitter and Facebook mention rates for articles on all disciplines. That is, for all disciplines, the newer *Nature* articles have relatively higher impact on Twitter and Facebook. For all articles published from 2010 to 2015, we also can see that the articles on biology sciences and earth & environment sciences have higher impact on Twitter and Facebook than the other two disciplines. Note that, for the papers published in 2015, the papers on earth & environment sciences have the highest impact on both Twitter and Facebook among all disciplines.

For a specific *Nature* article, there may be distinct impacts on Twitter for different Twitter user types. In order to evaluate impacts of articles on different disciplines for different user types, we study the Twitter mention rate by user type and discipline. As shown in Fig 2(d), it is the Twitter mention rate by user type and discipline. It can be found that for all disciplines, there is a highest impact on members of the public, and then on scientists, science communicators, and practitioners. For members of the public, scientists and science communicators, the impact of the articles on chemical sciences is much lower than those of the articles on other three disciplines. Moreover, for all disciplines, there is a relatively small impact on practitioners and science communicators.

To identify the highest social impact discipline and research field in *Nature* based on altmetrics, we analyze the most tweeted papers and the most posted *Nature* papers on Facebook. Table 2 shows the top fifteen most tweeted articles in *Nature*. Two articles were tweeted more than 3000 times, one article was tweeted between 2000 and 3000 times, and five articles were tweeted between 1000 and 2000 times. Ten of the fifteen most tweeted papers belong to biology sciences, and the others belong to physical sciences and earth & environment sciences. Many of these papers about human health (Rank 1 and 3), reprogramming (Rank 2), neuroscience (Rank 4 and 7), quantum (Rank 5 and 15), climatic variation (Rank 6 and 10), stem cell (Rank 8 and 13), computer science (Rank 9), synthetic biology (Rank 11), archaeology (Rank 12), and archaeal evolution (Rank 14). We also can find that most (12 of 15) of the highly tweeted articles were published in 2014 and 2015. That maybe because the public pays more attentions to the academic field in recent years with the increasing evolution of Twitter.

**Table 2:** Most Tweeted Papers in the *Nature*.

Rank	Title	Discipline	Keywords	Date	Tweets
1	Artificial sweeteners induce glucose intolerance by altering the gut microbiota	Biology sciences	Type 2 diabetes mellitus, Microbiome, Metabolic syndrome, Metagenomics	2014/9/17	3664
2	Stimulus-triggered fate conversion of somatic cells into pluripotency	Biology sciences	Reprogramming	2014/1/29	3442
3	A new antibiotic kills pathogens without detectable resistance	Biology sciences	Natural products, Target identification, Antibiotics, Antimicrobial resistance	2015/1/7	2466
4	Selective corticostriatal plasticity during acquisition of an auditory discrimination task	Biology sciences	Neuroscience	2015/3/2	1995
5	Observation of Dirac monopoles in a synthetic magnetic field	Physical sciences	Ultracold gases, Bose-Einstein condensates, Quantum fluids and solids	2014/1/29	1720
6	The geographical distribution of fossil fuels unused when limiting global warming to 2°C	Earth & environment sciences	Climate-change mitigation	2015/1/7	1387
7	Structural and functional features of central nervous system lymphatic vessels	Biology sciences	Neuroimmunology, Lymphatic vessels	2015/6/1	1169
8	Bidirectional developmental potential in reprogrammed cells with acquired pluripotency	Biology sciences	Stem cells	2014/1/29	1122
9	Human-level control through deep reinforcement learning	Physical sciences	Computer science	2015/2/25	962
10	No increase in global temperature variability despite changing regional patterns	Earth & environment sciences	Climate and Earth system modelling	2013/7/24	806
11	A semi-synthetic organism with an expanded genetic alphabet	Biology sciences	Synthetic biology, DNA metabolism, DNA replication	2014/5/7	768
12	3.3-million-year-old stone tools from Lomekwi 3, West Turkana, Kenya	Biology sciences	Archaeology	2015/5/20	761
13	Cerebral organoids model human brain development and microcephaly	Biology sciences	Biological models, Neurogenesis, Stem cells, Diseases of the nervous system	2013/8/28	738
14	Complex archaea that bridge the gap between prokaryotes and eukaryotes	Biology sciences	Archaeal evolution, Origin of life, Metagenomics	2015/5/6	709
15	Attractive photons in a quantum nonlinear medium	Physical sciences	Atomic and molecular interactions with photons, Nonlinear optics, Quantum optics, Quantum mechanics	2013/9/25	695

As given in Table 3, they are the top fifteen most posted *Nature* articles on Facebook. One paper was posted more than 300 times, one paper was posted between 200 and 300 times, and three papers were posted between 100 and 200 times. Twelve of the fifteen most posted articles belong to biology sciences, whereas the others belong to physical sciences and earth & environment sciences. We can find that the highly mentioned articles on Facebook are about human health (Rank 1, 3, 5, 10 and 13), archaeology (Rank 2), reprogramming (Rank 4), quantum (Rank 6 and 9), neuroimmunology (Rank 7), gene (Rank 8, 11 and 14), climatic variation (Rank 12), and psychology (Rank 15). Compared with Twitter, highly mentioned articles on Facebook are more about human health. Users of Twitter and Facebook both pay more attention to biology sciences. Moreover, there are 7 articles that are both most tweeted and posted on Facebook and these articles are related to biology science, physical sciences and earth & environment sciences.

**Table 3:** Most Posted *Nature* Papers on Facebook.

Rank	Title	Discipline	Keywords	Date	Facebook Posts
1	Artificial sweeteners induce glucose intolerance by altering the gut microbiota	Biology sciences	Type 2 diabetes mellitus, Microbiome, Metabolic syndrome, Metagenomics	2014/9/17	351
2	Homo erectus at Trinil on Java used shells for tool production and engraving	Biology sciences	Archaeology	2014/12/3	288
3	A new antibiotic kills pathogens without detectable resistance	Biology sciences	Natural products, Target identification, Antibiotics, Antimicrobial resistance	2015/1/7	156
4	Stimulus-triggered fate conversion of somatic cells into pluripotency	Biology sciences	Reprogramming	2014/1/29	145
5	Diet rapidly and reproducibly alters the human gut microbiome	Biology sciences	Microbiome, Inflammatory bowel disease	2013/12/11	121
6	Observation of Dirac monopoles in a synthetic magnetic field	Physical sciences	Ultracold gases, Bose-Einstein condensates, Quantum fluids and solids	2014/1/29	92
7	Structural and functional features of central nervous system lymphatic vessels	Biology sciences	Neuroimmunology, Lymphatic vessels	2015/6/1	82
8	Translating dosage compensation to trisomy 21	Biology sciences	Gene silencing, Gene therapy	2013/7/17	75
9	Attractive photons in a quantum nonlinear medium	Biology sciences	Atomic and molecular interactions with photons, Nonlinear optics, Quantum optics, Quantum mechanics	2013/9/25	71
10	Dietary emulsifiers impact the mouse gut microbiota promoting colitis and metabolic syndrome	Biology sciences	Microbial communities, Chronic inflammation	2015/2/25	68
11	The genomic signature of dog domestication reveals adaptation to a starch-rich diet	Biology sciences	Evolutionary genetics, Population genetics, Genomics, Metabolism	2013/1/23	63
12	The geographical distribution of fossil fuels unused when limiting global warming to 2°C	Earth & environment sciences	Climate-change mitigation	2015/1/7	63
13	Sodium chloride drives autoimmune disease by the induction of pathogenic TH17 cells	Biology sciences	Autoimmunity	2013/3/6	62
14	Towards practical, high-capacity, low-maintenance information storage in synthesized DNA	Physical sciences	DNA nanotechnology, Synthetic biology, Information technology, DNA and RNA	2013/1/23	58
15	Spontaneous giving and calculated greed	Biology sciences	Evolution, Psychology	2012/9/19	54

### Relationship Analysis between Tweets and Citations

The analysis for the relationship between tweets and citations can help us to determine whether tweets and citation measure similar concepts. Before assessing the relationship between tweets and citations for *Nature* articles, we first identify the most cited papers in *Nature*. Table 4 shows the top fifteen most cited *Nature* papers published from 2010 to 2015. One article was cited more than 2000 times, and ten article was cited between 1000 and 2000 times. Twelve of the high-impact papers belong to biology sciences, whereas the others belong to physical sciences. Many of these papers are about genetics (Rank 1-5, 7, and 9-13), solar energy (Rank 6), structural biology (Rank 8), astronomy (Rank 14) and materials science (Rank 15). Different from Twitter and Facebook, all the highly cited articles were published before 2014, because the citation needs time to accrue and social medias are real-time. In addition, it is likely social users and researchers focus on different academic points.

**Table 4:** Most Cited Papers in the *Nature*.

Rank	Title	Discipline	Keywords	Date	Cites	Tweets
1	An integrated encyclopedia of DNA elements in the human genome	Biology sciences	Genetics, Genomics, Molecular biology	2012/9/5	2177	272
2	An integrated map of genetic variation from 1,092 human genomes	Biology sciences	Genetics, Genomics	2012/10/31	1479	570
3	Comprehensive molecular portraits of human breast tumours	Biology sciences	Cancer, Genomics, Molecular biology, Genetics	2012/9/23	1455	238
4	Mammalian microRNAs predominantly act to decrease target mRNA levels	Biology sciences	Molecular biology, Genetics, Genomics	2010/8/15	1398	3
5	Biological, clinical and population relevance of 95 loci for blood lipids	Biology sciences	Genetics, Genomics	2010/8/8	1174	3
6	Sequential deposition as a route to high-performance perovskite-sensitized solar cells	Physical sciences	Solar cells, Solar energy and photovoltaic technology, Synthesis and processing, Design, synthesis and processing	2013/7/10	1099	20
7	Enterotypes of the human gut microbiome	Biology sciences	Genetics and genomics	2011/4/20	1080	53
8	Crystal structure of oxygen-evolving photosystem II at a resolution of 1.9 Å	Biology sciences	Structural biology, Plant sciences, Biophysics, Biochemistry	2011/4/17	1055	9
9	Integrated genomic analyses of ovarian carcinoma	Biology sciences	Cancer, Genetics, Genomics	2011/6/29	1053	38
10	The zebrafish reference genome sequence and its relationship to the human genome	Biology sciences	Comparative genomics	2013/4/17	1046	194
11	Comprehensive molecular characterization of human colon and rectal cancer	Biology sciences	Cancer, Genomics, Genetics, Health and medicine	2012/7/18	1022	164
12	Global quantification of mammalian gene expression control	Biology sciences	Cell biology, Molecular biology, Biotechnology, Genetics, Genomics	2011/5/18	967	12
13	Structure, function and diversity of the healthy human microbiome	Biology sciences	Ecology, Microbiology, Genetics, Genomics, Health and medicine	2012/6/13	940	159
14	A two-solar-mass neutron star measured using Shapiro delay	Physical sciences	Astronomy, Astrophysics	2010/10/27	883	4
15	Atomically precise bottom-up fabrication of graphene nanoribbons	Physical sciences	Materials science	2010/7/25	881	0

To explore the relationship between tweets and citations for *Nature* articles, we analyze the Spearman correlation between tweets and citations at the article level like other researches [20, 27] did. Correlations with a coefficient smaller than 0.30, between 0.30 and 0.50, and larger than 0.50 are considered weak, moderately strong and strong, respectively [28]. Note that, as shown in Fig 1(b), for all disciplines, the Twitter coverage for *Nature* papers published in 2010 is less than 40%, which is too low to analyze the relationship based on Spearman correlation. Thus, we mainly focus on the correlation analysis result about the articles published from 2011 to 2014, while the result about the articles published in 2010 is for reference only.

Table 5 shows the Spearman correlations between tweets and citations for *Nature* articles published in different years in terms of disciplines. We can find that there is a relatively higher positive correlation for papers on all disciplines published in 2012. Moreover, the correlation for the articles on biology sciences and earth & environment sciences is positive and significant with 1% significance level. Recall that articles on biology sciences and earth & environment sciences have higher Twitter mention rate according to Fig 2(b). So an interesting discovery is rised that there is a relatively large positive correlation for the discipline with high Twitter mention rate. It should be noted that the correlation for the papers on chemical sciences published in 2014 is negative, but it is insignificant. For physical sciences, the correlations are positive but very low in general compared with the other three disciplines. Moreover, for the articles published from 2011 to 2014, the correlation coefficient shows first increasing then decreasing as the publication time passed. The reason for this seems to be the development of Twitter and the time-delay of citation. In the early days of Twitter, the limited Twitter user number leads to a low correlation between tweets and citations. Then, the development of internet triggers a blossom of Twitter users, and more researchers discuss and diffuse academic information on Twitter, so the correlation is increasingly strong over time. Moreover, since the citation counts of articles published in recent years are unstable and incomplete due to the citation delay, the correlation reduces for the articles published after 2012. This finding suggests that the relationship analysis between tweets and citations can be biased by changes in Twitter use and citation delays.

**Table 5:** Spearman correlation between tweets and citations.

	2010	2011	2012	2013	2014
Biology Sciences	0.258**	0.153**	0.366**	0.365**	0.181**
Chemical Sciences	0.467**	0.065	0.313**	0.35	**-0.22**
Earth & Environment Sciences	0.23*	0.298**	0.42**	0.419**	0.301**
Physical Sciences	0.06	0.022	0.233**	0.122	0.017
Total	0.259**	0.161**	0.354**	0.321**	0.16**

* Correlation is significant at the 0.05 level.

** Correlation is significant at the 0.01 level.

As shown in Table 6, it is the Spearman correlation between tweets and citations by Twitter user types. We can find that for all user types, the correlation of the articles published in 2012 and 2013 is relatively higher compared with the articles published in others three years. From the perspective of discipline, except chemical sciences, the correlation for articles on other three disciplines is higher for the user type of scientist (the highest correlation coefficients of biology sciences, earth & environment sciences, and physical sciences are 0.389, 0.424, and 0.519, respectively). Citations of articles on chemical sciences have a high correlation with tweets of science communicator (the highest correlation coefficients of chemical sciences is 0.519). From the angle of Twitter user type, for member of the public and scientist, their tweets are highly correlated with the citations of articles on biology sciences and earth & environment sciences than the other two disciplines. The tweets of practitioners are highly correlated with the citations of articles on biology sciences. We also can see that for science communicators, there is no significant correlation between their tweets and citations for articles on physical sciences. Note that, the Twitter coverage of some user types below 20 percent is too low to analyze the relationship based on Spearman correlation, and the related result is for reference only. Above all, the Twitter user type and the discipline have a great influence on correlation between tweets and citations. Therefore, scientometricians should consider the effect of Twitter user type and discipline when using altmetrics to rank articles.

**Table 6:** Spearman Correlation between Tweets and Citations by Twitter User Type.

		Biology Sciences		Chemical Sciences		Earth & Environment Sciences		Physical Sciences		Total	
		Correlation	Coverage	Correlation	Coverage	Correlation	Coverage	Correlation	Coverage	Correlation	Coverage
M. of P.	2010	0.184**	0.295	0.292**	0.149	0.151	0.231	0.106	0.195	0.205**	0.260
	2011	0.153**	0.739	0.073	0.634	0.293**	0.717	0.026	0.616	0.158**	0.692
	2012	0.324**	0.976	0.289**	0.970	0.404**	0.920	0.213**	0.912	0.318**	0.960
	2013	0.326**	0.995	0.416*	1.000	0.396**	0.979	0.144	0.956	0.299**	0.983
	2014	0.164**	0.995	-0.242	1.000	0.269**	1.000	0.018	0.994	0.132**	0.872
Sci.	2010	0.258**	0.210	0.366**	0.129	0.294**	0.179	0.068	0.150	0.239**	0.194
	2011	0.138**	0.681	0.06	0.627	0.238*	0.593	0.053	0.603	0.145**	0.635
	2012	0.389**	0.935	0.235**	0.879	0.424**	0.920	0.219**	0.749	0.367**	0.897
	2013	0.373**	0.939	0.2	0.840	0.379**	0.948	0.046	0.850	0.306**	0.916
	2014	0.211**	0.930	0.023	0.813	0.301**	0.979	0.006	0.867	0.185**	0.830
Pra.	2010	0.177**	0.055	0.156	0.030	0.095	0.167	0.001	0.027	0.136**	0.045
	2011	0.231**	0.199	0.084	0.093	0.363**	0.097	0.029	0.075	0.248**	0.158
	2012	0.340**	0.421	0.207*	0.205	0.123	0.232	0.074	0.175	0.314**	0.348
	2013	0.348**	0.488	-0.111	0.160	0.14	0.229	0.006	0.231	0.315**	0.405
	2014	0.171**	0.513	0.174	0.250	0.246*	0.284	0.153*	0.376	0.185**	0.463
S. C.	2010	0.023	0.055	0.115	0.020	0.195	0.064	0.208*	0.027	0.075	0.044
	2011	0.061	0.220	0.146	0.149	0.157	0.265	0.033	0.212	0.062	0.214
	2012	0.254**	0.615	0.255**	0.424	0.306**	0.625	0.144	0.392	0.245**	0.567
	2013	0.321**	0.628	0.519**	0.360	0.39**	0.604	0.2*	0.481	0.292**	0.585
	2014	0.169**	0.721	0.014	0.438	0.166	0.758	0.07	0.564	0.145**	0.597

* Correlation is significant at the 0.05 level.

** Correlation is significant at the 0.01 level.

M. of P. is member of the public. Sci. is scientists. Pra. is practitioner. S. C. is science communicator.

## Conclusion

In this paper, we have assessed the *Nature* publications over the period between January 2010 and June 2015 based on altmetrics. Firstly, we examine the representativeness and validity of Twitter and Facebook as a source of altmetrics based on the distribution of *Nature* papers on Twitter and Facebook. The increase of coverage and mention rate over publication date shows the development of social media platforms makes people more aware of academic findings. There are obvious differences between different social media platforms on the social concern degree and impact of scholarly papers based on the comparative analysis for the coverage and mention rate of Twitter and Facebook. Social concern degree and impact of scholarly papers on Twitter are higher and have a faster growth rate than Facebook.

Moreover, the people’s concerns on different disciplines are very different. While the general public pay more attention to the papers related to their daily lives such as health and climatic variation, unsurprisingly we observe that the general public express more interests in papers on biology sciences and earth & environment sciences according to our analysis of the top fifteen most tweeted articles and posted articles on Facebook. In addition, all user types of Twitter are found to share much more interests in biology sciences than other disciplines, although different user types of Twitter show different concerns for different disciplines according to the analysis of coverage and mention rate over Twitter user types. According to the distribution analysis, Twitter is found to be more representative and valid as a source of altmetrics than Facebook.

Secondly, we explore the correlation between tweets and citations. The correlation between tweets and citations for *Nature* articles is positive and appears quite sensitive to the publication date, discipline and Twitter user type. The variation tendency of correlation coefficient by publication date suggests that the relationship between tweets and citations is influenced by changes in Twitter use and citation delays. As shown in Tables 5 and 6, all significant correlation coefficients are less than 0.52. This implies that although tweets and citations are somewhat related, they mostly measure different types of impact. The tweets of the top fifteen most cited articles are lower than the tweets shown on Table 2 and this also shows tweets and citations are different impact evaluation index. In addition, for the analysis of the correlation between tweets and citations, we consider the impact of Twitter coverage on the validity of the results because the lower Twitter coverage can lead to some deviations.

Overall, our research presents a generic analysis of representativeness and validity of altmetrics data sources and relation between citation and altmetrics based on discipline, publications date and Twitter user type. Our results provide a new reference for the development of subsequent research in altmetrics.

This study is limited by the integrity of data (we crawl data from the internet), and comprehensiveness of analysis (the analysis just covers two social media platforms and one journal). Hence, further research could include the systematic analysis of all social media platforms based on more authoritative data. This will help determining whether the altmetrics indicators complements to traditional metrics in research impact assessment.

## Supporting Information

S1 File Information of Nature papers. We crawled data from Web of Science (https://login.webofknowledge.com) and Nature (http://www.nature.com). We obtained the title, discipline, keywords, doi, published time, tweets and facebook posts from Nature, doi and citations from Web of Science. These data are combined by doi to support our analysis. There is some null information in our file because the data are crawled from the internet. But we do some fault-tolerant processing to ensure the accuracy of our analysis such as utilising the average.
(XLSX)
